# Irreversible AE1 Tyrosine Phosphorylation Leads to Membrane Vesiculation in G6PD Deficient Red Cells

**DOI:** 10.1371/journal.pone.0015847

**Published:** 2011-01-05

**Authors:** Antonella Pantaleo, Emanuela Ferru, Franco Carta, Franca Mannu, Luigi F. Simula, Amina Khadjavi, Proto Pippia, Francesco Turrini

**Affiliations:** 1 Department of Genetics, Biology and Biochemistry, University of Turin, Turin, Italy; 2 Section of Internal Medicine, Department of Clinical and Experimental Medicine, University of Verona, Verona, Italy; 3 Nurex S.r.l., Sassari, Italy; 4 Hospital of Alghero, ASL 1- Sassari, Sassari, Italy; 5 Department of Physiological, Biochemical and Cell Sciences, University of Sassari, Sassari, Italy; University of Cambridge, United Kingdom

## Abstract

**Background:**

While G6PD deficiency is one of the major causes of acute hemolytic anemia, the membrane changes leading to red cell lysis have not been extensively studied. New findings concerning the mechanisms of G6PD deficient red cell destruction may facilitate our understanding of the large individual variations in susceptibility to pro-oxidant compounds and aid the prediction of the hemolytic activity of new drugs.

**Methodology/Principal Findings:**

Our results show that treatment of G6PD deficient red cells with diamide (0.25 mM) or divicine (0.5 mM) causes: (1) an increase in the oxidation and tyrosine phosphorylation of AE1; (2) progressive recruitment of phosphorylated AE1 in large membrane complexes which also contain hemichromes; (3) parallel red cell lysis and a massive release of vesicles containing hemichromes. We have observed that inhibition of AE1 phosphorylation by Syk kinase inhibitors prevented its clustering and the membrane vesiculation while increases in AE1 phosphorylation by tyrosine phosphatase inhibitors increased both red cell lysis and vesiculation rates. In control RBCs we observed only transient AE1 phosphorylation.

**Conclusions/Significance:**

Collectively, our findings indicate that persistent tyrosine phosphorylation produces extensive membrane destabilization leading to the loss of vesicles which contain hemichromes. The proposed mechanism of hemolysis may be applied to other hemolytic diseases characterized by the accumulation of hemoglobin denaturation products.

## Introduction

G6PD deficiency affects more than 400 million people worldwide, with a prevalence varying from 10 to 25% in most areas where malaria is endemic. This genetic defect provides partial protection against malaria, but may lead to severe hemolytic episodes after the administration of some drugs (anti-malarials, anti-inflammatories, vitamin K, etc.), the ingestion of fava beans (favism) or infection [1]–[3]. Typically the appearance of the first symptoms occurs 24–48 hours after the intake of pro-oxidant drugs or fava beans.

While the molecular biology of G6PD deficiency has been extensively studied [2], the molecular mechanisms leading to the hemolytic crisis are still unclear. G6PD deficient red cells (G^−^ RBCs) display a failure of the protective response to oxidant stress, which leads to irreversible oxidation of glutathione [1], [2], [4]–[6]. The accumulation of large hemichrome aggregates (Heinz bodies) is an additional hallmark of the hemolytic crisis in G^−^ individuals [7].

Some membrane alterations have been described in G^−^ RBCs, such as the oxidation and clustering of membrane proteins, the binding of hemichromes to the internal face of the membrane, the destabilization of the membrane and the release of micro-vesicles [8]–[10]. Interestingly, increased hemichrome formation has been observed in G^−^ RBCs infected by malaria parasites [11]. The data available on membrane modifications are in any case insufficient to formulate a clear hypothesis as to the mechanisms of membrane destabilization and G^−^ RBC destruction. The dearth of information concerning the mechanisms of red cell lysis represents a practical drawback which impedes both any prediction about the hemolytic activity of drugs and the understanding of the large individual susceptibility even in presence of the same G6PD mutation [1].

The authors, as well as others have shown that band 3 red cell membrane protein (AE1) displays a marked tendency to become tyrosine phosphorylated in G- RBCs after –SH group oxidation or GSH depletion by 1-chloro-2,4-dinitrobenzene (CDNB) or diamide [12], [13]. We have also demonstrated that Syk tyrosine kinase strongly increases its affinity to oxidized AE1 and induces its selective phosphorylation [13]. Hyper-phosphorylated AE1 showed a manifest tendency to cluster, indicating a change in its interactions with the cytoskeletal network. Furthermore, abnormal AE1 tyrosine phosphorylation has been observed in a number of red cell disorders [14].

In the present study we have demonstrated that following –SH group oxidation induced by diamide (–SH group oxidant) and divicine, an oxygen reactive compound held responsible for favism [15], AE1 becomes increasingly and irreversibly phosphorylated in G^−^ RBCs. Syk kinase inhibition largely prevents red cell membrane lysis and vesiculation, strongly suggesting a functional role of AE1 tyrosine phosphorylation in the red cell membrane destabilization.

## Results

### Short and long term effects of oxidants in G6PD deficient red cells

Previous work has described how oxidant treatments induce more intense AE1 tyrosine phosphorylation in G^−^ RBCs than in control RBCs [13], [14], [16]. In the present study, we analyzed AE1 phosphorylation and a series of additional parameters for longer time exposure with diamide, an -SH group oxidant reagent, or with divicine [15], a compound extracted from fava beans considered responsible for severe hemolytic crises in G^−^ deficient subjects [5], [15]. The long term effects of oxidants in the G^−^ RBCs were not easily predictable as, although the G^−^ RBC samples used in our experiments had low G6PD levels (Mediterranean variant 563 C —> T with approximately 2–3% of normal red cell G6PD activity level), the hexose monophosphate shunt activity in these cells presented normal activity and could be 2–3 fold further activated following oxidant treatments [17].

**Figure 1:**
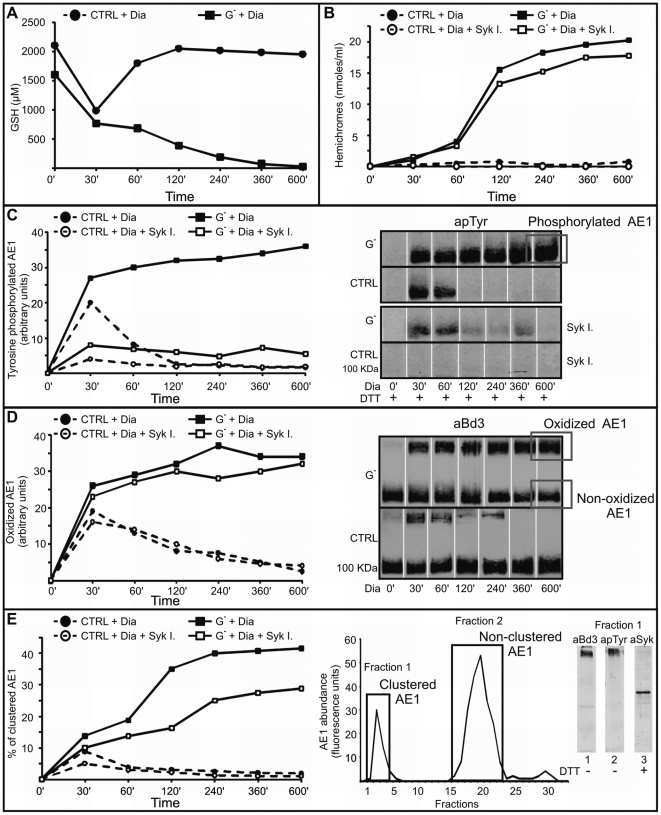
Time course of AE1 tyrosine phosphorylation, oxidation and clustering. GSH levels measured in control (CTRL) and G6PD deficient (G^−^) RBCs upon diamide treatment, expressed as µM (Fig. 1A). Amounts of hemichromes (HMC) measured in isolated membranes of control and G6PD deficient RBCs (Fig. 1B). HMCs were quantified by Vis spectrometry and expressed as nmoles/ml. Quantitative densitometry of AE1 phosphorylation levels (Fig. 1C) and of the oxidatively cross-linked AE1 (Fig. 1D) in control and G^−^ RBCs. Quantification of AE1 phosphorylation levels and of oxidized AE1 was performed with an IR fluorescence detection scanner (Odyssey, Licor, USA) of anti-phosphotyrosine (apTyr) and anti-AE1 (aBd3) western blots with Odyssey V3.0 software and expressed as fluorescence arbitrary units. The western blots in figure 1C and 1D show the areas used for AE1 tyrosine phosphorylation and oxidation quantifications. Membrane proteins were solubilized in presence (Fig. 1C) or absence (Fig. 1D) of the reducing agent (DTT). Percentages of clustered AE1 after gel filtration separation of the high molecular weight protein complexes (Fig. 1E). Membrane proteins were extracted from control and G6PD deficient RBCs by 1% triton-X100, the supernatant was applied to a 40×1 cm column filled with Sepharose CL-6B to isolate the high molecular weight membrane protein complexes. AE1 was quantified by eosine maleimide fluorescence detection. Clustered AE1 was quantified as percentage of total AE1 eluted from the column. The chromatogram in figure 1E shows the two peaks corresponding to clustered and non-clustered AE1. The western blots in figure 1E show the presence of aggregated AE1 (aBd3), its tyrosine phosphorylation (apTyr) and Syk (aSyk) in the high molecular weight fraction. Membrane proteins were solubilized in absence (lanes 1 and 2) or in presence (lane 3) of the reducing agent (DTT). Control and G6PD deficient RBCs were treated with 0.25 mM diamide (Dia) in presence or absence of Syk inhibitors 10 µM (Syk I.) at different incubation times (0–600 minutes). Values are means of 3 experiments. All differences observed between control and G6PD deficient RBCs were significant (p<0.01). In Figure 1C and 1E (only in G6PD deficient and after 60 minutes) the changes caused by Syk inhibitors were significant (p<0.01).

Following 0.25 mM treatment with diamide, approximately 80% of reduced glutathione (GSH) was oxidized within 5 minutes and the pre-treatment levels were restored within 45 minutes in control RBCs. Conversely, in G^−^ RBCs reduced GSH further declined and reached un-measurable levels within 2 hours of incubation (Fig. 1A). Indistinguishable GSH response was elicited by divicine 0.5 mM. On the basis of these results we decided to use these concentrations both for diamide and divicine.

Figure 1C shows that following oxidant treatment, in G^−^ RBCs, AE1 phosphorylation progressively increased during the course of incubation (10 hours). In control RBCs AE1 phosphorylation was completely reverted in approximately 1 hour. Syk kinase inhibitors markedly reduced the rate of AE1 phosphorylation both in control and G^−^ RBCs. AE1 oxidation (disulfide cross-linking) parallels its tyrosine phosphorylation both in control and G^−^ RBCs (Fig. 1D). As expected Syk inhibitors did not exert an apparent effect on AE1 oxidation.

Figure 1E shows that following oxidant treatment, in G^−^ RBCs, AE1 was increasingly recruited in a high molecular weight cluster [13], [18], while in control RBCs, AE1 clustering showed transient behavior. AE1 large cluster formation appeared to be delayed in comparison to the AE1 phosphorylation and oxidative cross-linking. Syk kinase inhibitors decreased the amount of clustered AE1. Analysis of the clustered AE1 fraction revealed that AE1 was prevalently oxidized, cross-linked and phosphorylated. Anti-Syk western blot revealed a time dependent increase of Syk associated with the high molecular weight fraction. In the same fraction we observed the presence of hemoglobin denaturation products (see below).

Figure 1B shows that the hemichrome quantities measured in isolated RBC membranes increased with time after oxidant treatment of G^−^ RBCs. In control RBCs no hemichrome formation was observed. Syk inhibitors did not exert an apparent effect on hemichrome formation. Measuring the hemichromes also in the high molecular weight clusters, we have estimated that approximately 75% of hemichromes were associated with this membrane protein complex.

At the chosen concentrations, diamide and divicine caused very similar effects in all monitored parameters indicating that the long term effects of divicine are probably triggered by sulfidril groups oxidation.

In conclusion, this set of results indicates that even after long term incubation, G^−^ RBCs are unable to restore the initial levels of GSH and, consequently, to reduce the disulfide bonds of AE1. In G^−^ RBCs, AE1 tyrosine phosphorylation and clustering also increase with time after oxidant treatment. Syk inhibitors cause a marked reduction of both AE1 phosphorylation and clustering without effecting its oxidative cross-linking and hemichrome formation, suggesting that persistent AE1 phosphorylation plays a role in the formation of the large membrane complexes.

### Effect of Syk kinase and PTP inhibitors on G6PD deficient red cell lysis caused by oxidant treatments

**Figure 2:**
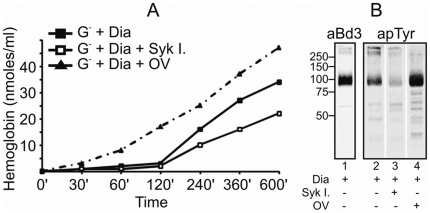
Time course of G6PD deficient RBC lysis. A. Quantification of haemoglobin released from G6PD deficient (G^−^) RBCs that were treated with 0.25 mM diamide (Dia) in presence or absence of Syk inhibitors 10 µM (Syk I.) and o-vanadate 1 mM (OV) at different incubation times (0–600 minutes). Values are means of 3 experiments and are expressed as nmoles/ml. The changes caused by Syk inhibitors (after 120 minutes) and OV (after 60 minutes) were significant (p<0.01). B. Anti band 3 (aBd3) and anti-phosphotyrosine (apTyr) western blots of G^−^ RBCs treated with diamide 0.25 mM (lanes 1 and 2) in absence or presence of Syk inhibitors (lane3) or o-vanadate (lane 4). Membrane proteins were solubilized in presence of the reducing agent (DTT).

Observing the supernatant of G^−^ RBC culture we noticed a marked lysis some hours after the exposure to oxidants. No hemolysis was observed in control RBCs. Figure 2A shows that red cell lysis became evident after 2–3 hours of incubation in G^−^ RBCs, increasing progressively with the time. Pre-treatment with Syk kinase inhibitors caused a consistent reduction of the hemolysis, while phosphatase inhibition by o-vanadate caused an accelerated and more intense hemoglobin leakage indicating an association between G^−^ RBCs lysis and AE1 phosphorylation levels (Fig. 2B).

**Figure 3:**
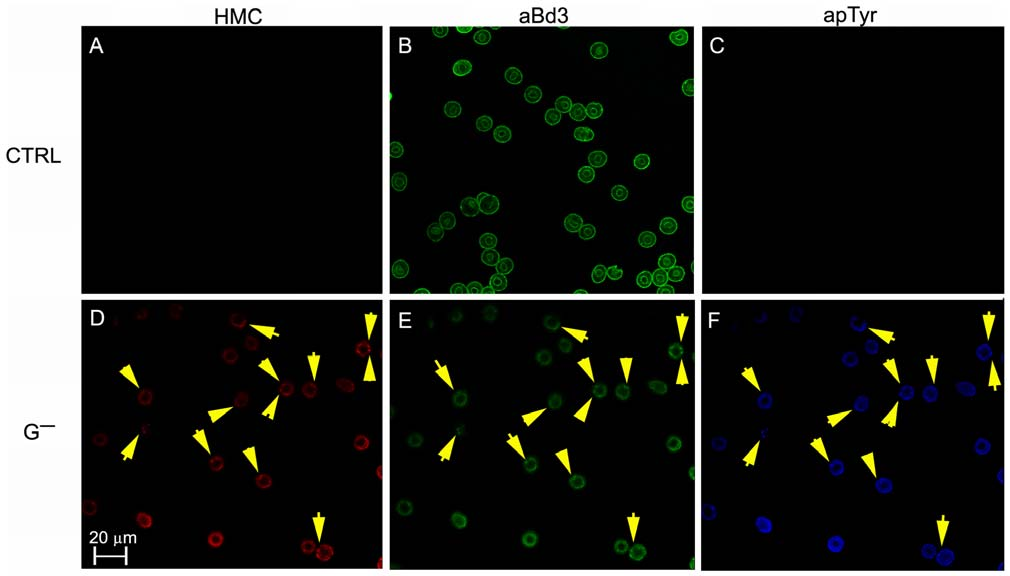
Confocal microscopy images of control and G6PD deficient RBCs. Panels A D: hemichrome (HCM) autofluorescence; Panels B, E staining with anti-band 3 antibody (aBd3); Panels C, F staining with anti-phosphotyrosine antibody (apTyr). Panels A, B, C control RBCs (CTRL). Panels D, E, F G6PD deficient red cells (G^−^). Yellow arrows indicate clusters containing hemicromes and co-stained with anti AE1 and anti-phosphotyrosine antibodies. Bar: 20 µm.

Confocal microscopy analysis revealed that oxidant exposure induced marked changes exclusively in G^−^ RBCs. Following a 2 hour incubation with diamide 0.25 mM, hemichrome formation was already observable: staining with both AE1 and anti-phosphotyrosine antibodies revealed an uneven membrane distribution and an apparent co-association with the hemichrome clusters (Fig. 3). Hemichromes and tyrosine phosphorylation were not observed in control RBCs treated in the same conditions.

### Characterization of membrane vesicles released from G6PD deficient red cells following oxidant treatments

**Figure 4:**
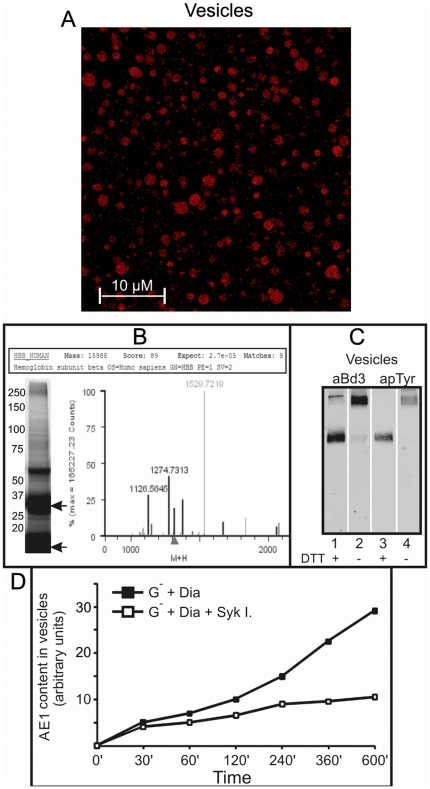
Vesicle characterization. A. Confocal image of isolated vesicles using hemichrome auto-fluorescence (excitation 488 nm, emission 630–750 nm). Bar: 10 µm. B. SDS PAGE of isolated vesicles and mass spectrometry identification of haemoglobin in the two most intense gel bands (indicated by the arrows). C. Corresponding western blot with anti AE1 (aBd3) (lanes 1 and 2) and anti-phosphotyrosine (apTyr) (lanes 3 and 4) antibodies under reducing (DTT) and non reducing conditions. D. Quantification of AE1 contained in vesicles released from G6PD deficient RBCs (G^−^). Quantification of AE1 was performed by IR fluorescence detection of anti-phosphotyrosine and anti-band 3 western blots (Odyssey, Licor, USA) with Odyssey V3.0 software and expressed as fluorescence arbitrary units. RBCs were treated with 0.25 mM diamide (dia) in presence or absence of Syk inhibitors 10 µM (Syk I.) at different incubation times (0–600 minutes). Values are means of 3 experiments. The changes caused by Syk inhibitors (after 120 minutes) were significant (p<0.01).

Vesicles were isolated from red cell cultures (4 hours of incubation with diamide 0.25 mM) by ultra-centrifugation and then characterized by confocal analysis of hemichrome auto-fluorescence, western blotting and mass spectrometry. Confocal microscopy analysis was suggestive of large amounts of hemichromes in isolated vesicles (Fig. 4A). The presence of hemichromes in isolated vesicles was further demonstrated by Vis spectrometry analysis indicating that a large fraction (approximately 90%) of hemoglobin contained in the vesicles, was present in the form of hemichromes (data not shown). SDS-PAGE separation of vesicle proteins and the identification by mass spectrometry of the more prominent bands, further supported the presence of large amounts of hemoglobin products (Fig. 4B). Western blotting analysis of isolated vesicles demonstrated that they contained oxidized, cross-linked and phosphorylated AE1 (Fig. 4C). The quantitative measurement of AE1 evidenced the time course of vesicle release. A marked reduction of vesicle release was observed following Syk inhibitor treatment (Fig. 4D).

## Discussion

In a previous study [13] we observed that AE1 phosphorylation was apparently more pronounced and long standing in G^−^ RBCs than in control RBCs following their treatment with diamide but, after a short incubation time, we did not observe any functional consequence. Hemolytic crises in G^−^ subjects occur many hours after the intake of pro-oxidant compounds [7], therefore, in the present study we investigated the long term effects (up to 10 hours) of the treatment of G^−^ RBCs with diamide (an -SH group oxidant reagent) or divicine (a compound extracted from fava beans held responsible for severe hemolytic crises in G^−^ deficient subjects) [15].

The treatment with these compounds caused similar short term effects in G^−^ and control RBCs but after 2–3 hours of incubation, striking differences were observed between the two types of red cells. While control RBCs returned to the pre-treatment conditions, G^−^ RBCs showed a time dependent modification of several parameters: i) the GSH levels and -SH groups of AE1 continued to decrease; ii) Syk tyrosine kinase became irreversibly associated with the membrane; iii) AE1 tyrosine phosphorylation steadily increased over time, iv) hemichromes became progressively bound to the membrane; v) phosphorylated AE1 formed large clusters; vi) these clusters were exovesiculated and this phenomenon was associated with lysis.

We have observed that Syk inhibitors consistently reduced the amount of vesiculation and lysis. The protective effect of Syk inhibitors suggested that, in G^−^ RBCs, AE1 hyper-phosphorylation may play a role in the progressive membrane damage induced by oxidant treatments.

This hypothesis is supported by some additional findings: i) o-vanadate (tyrosine phosphatase inhibitor) further increased AE1 phosphorylation and the rate of vesicle release, ii) we have isolated membrane protein complexes which contain both hemichromes and phosphorylated AE1, iii) confocal microscopy confirmed the co-association between hemichromes and phosphorylated AE1, iv) vesicles isolated from G^−^ RBCs revealed their high content of hemichromes and phosphorylated AE1.

The present findings are in accordance with previous results which have demonstrated that Syk kinase binds preferentially to oxidized AE1, inducing its tyrosine phosphorylation [13]. Increased red cell membrane fragility has often been associated with membrane vesiculation in different hematological situations [19]–[21]. It is interesting to note that the clustering of AE1 through its binding to hemichromes has been demonstrated in most of these conditions [22]–[29]. Moreover, in hemolytic diseases, the accumulation of vesicles derived from the red cell membranes has received particular consideration because of their potential pro-coagulant and pro-inflammatory activities [30], [31]. No direct data about vesicle accumulation in the plasma of G^−^ subjects undergoing hemolysis are currently available, but the presence of high levels of hemoglobin in the plasma strongly suggests the occurrence of intravascular hemolysis due to red cell membrane instability [7].

**Figure 5:**
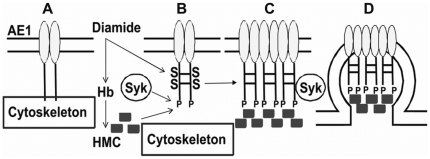
Schematic representation of the proposed mechanism. AE1 is dynamically associated with the cytoskeleton through ankyrin binding in untreated red cells (A). In G6PD deficient RBCs diamide causes irreversible AE1 disulfide cross-linking and its phosphorylation by Syk kinase, diamide also causes progressive hemichrome formation. In control RBCs AE1 oxidation and phosphorylation are transient and no hemichrome formation is observed (B). Hemichromes bind to AE1 and promote the clustering of phosphorylated AE1 (C). Large aggregates of AE1 and hemichromes are released in vesicles (D).

In conclusion, the present study contributes to the understanding of the mechanism of membrane destabilization and vesicle release occurring after the treatment of G^−^ RBCs with oxidant compounds (Fig. 5). As malaria parasites have been demonstrated to exert both oxidative damage and the binding of hemichromes to the red cell membrane [23], [32] it will be of great interest to verify whether the membrane alterations described herein may also be promoted by malaria parasites. In this case, the data presented may contribute to an explanation of the mechanism of malaria protection in G6PD deficient subjects.

AE1 clusters are known to bind naturally occurring antibodies and to trigger red cell phagocytosis [33], [34]. These phenomena may enhance the removal of vesicles which contain hemichromes, however, the role of antibodies and splenic macrophages has yet to be elucidated. In this respect, the functional meaning of the proposed mechanism in the selective removal of hemichromes from red cell membrane Vs the induction of membrane instability responsible for red cell lysis is to be clarified.

Additional studies will be required to further define a series of events such as: the irreversibility of AE1 tyrosine phosphorylation; the relative contributions of Syk kinase activation [12], [35]–[37] and tyrosine phosphatase inhibition [38]–[40]; the changes of AE1 – cytoskeleton interactions induced by AE1 phosphorylation; and the effect of additional membrane protein modifications [33].

## Materials and Methods

### Treatment of RBCs

Venous blood was drawn from 5 healthy and 5 G6PD deficient volunteers (Mediterranean variant 563 C —> T with approximately 2–3% of normal red cell G6PD activity level). Written informed consent was obtained from each blood donor to allow: “The use of 10 ml of their blood donation for research use. In particular, to study the causes of hemolytic episodes in G6PD deficient subjects”. Ethical approval to perform the present study was obtained from the “Ethical Committee of the ASL 1 – Sassari”. The data were analyzed anonymously and all clinical investigation was conducted according to the principal expressed in the Declaration of Helsinki. None of the donors showed clinical and laboratory evidence of hemolysis and all presented hemoglobin levels within the normal range. G6PD deficiency assessment was performed as previously described [22]. Red cells were pelleted at 1,000× g for 10 min at room temperature. After removal of the buffy coat, red blood cells (RBCs) were again pelleted and washed 3× with phosphate buffer saline (137 mM NaCl, 2.7 mM KCl, 8.1 mM K_2_HPO_4_, 1.5 mM KH_2_PO_4_, pH 7.4) in presence of 5 mM glucose (PBS-glucose) to obtain packed cells. To simulate oxidative stress, RBCs at 30% hematocrit were treated for different incubation times (0–600 min) at 37°C in PBS-glucose containing 0.25 mM of diamide (Sigma) or divicine (Serva, Heidelberg). Divicine was activated by β-glucosidase treatment as previously described [5].

For the inhibition of tyrosine phosphatases, RBCs were suspended at 30% hematocrit in PBS-glucose and incubated with 1 mM of o-vanadate in presence or absence of oxidants.

For the inhibition of Syk kinase, RBCs were suspended at 30% hematocrit in PBS-glucose and pre-incubated with 10 µM Syk inhibitors II and IV (Calbiochem) for 1 h at 37°C before oxidant treatments. The inhibitors were not washed prior to the oxidant treatment.

Each reaction was stopped by washing 3× in PBS-glucose, and membranes were prepared as described below. For all the protocols described above, untreated controls were processed identically, the only difference being the deletion of the stimulant/inducer from the incubation.

### RBC membrane preparation

Standard hypotonic membranes were prepared at 4°C on ice as previously described [18]. Briefly: 150 µL of packed RBCs were diluted into 1.5 mL of cold hemolysis buffer (5 mmol/L sodium phosphate, 1 mmol/L EDTA, pH 8.0) containing a protease and phosphatase inhibitor cocktail (Sigma-Aldrich, St.. Louis, MO) and then washed up to 4 more times in the same buffer (until membranes became white) in a refrigerated Eppendorf microfuge at 25,000× g. The preparations were stored frozen at −20°C until use. Membrane protein content was quantified using the DC Protein Assay (Biorad).

### Vesicle isolation

The supernatants of the samples treated as described above, were collected and centrifuged at 25000× g for 10 min at 4°C to eliminate spontaneously formed red cell ghosts. Lysis was quantified by measuring hemoglobin absorbance at 405 nm and expressed in nmoles/ml. After the addition of phosphatase infhibitors, supernatants were centrifuged for 3 hours at 100000× g on a refrigerated ultracentrifuge (Beckman) to isolate vesicles. Protein vesicles were identified by MALDI TOF MS [41].

### SDS-PAGE of membrane proteins

Membrane proteins were solubilized in Laemmli Buffer [42] under reducing (2% DTT) or non-reducing conditions in a volume ratio of 1∶1. SDS-PAGE analysis was conducted by heating the samples for 5 min at 100°C and loading 20 µg membrane proteins on the 8% gel for protein staining by blue colloidal Coomassie [43].

### Immunoblot analysis and IR fluorescence detection

Proteins separated by SDS/PAGE were transferred to nitrocellulose membranes as previously described [13] and then probed with either anti-phosphotyrosine antibody (Santa Cruz, CA) diluted to 1∶2000, or with anti-band 3 (AE1) antibody (Sigma Aldrich) diluted to 1∶50000. Secondary antibodies conjugated to infrared fluorescent dyes excitable at 800 nm (IRDye 800CW, Li-COR-USA) were then used to visualize the desired antigens using an 800 nm laser scanner (Odyssey, Licor, USA). To establish the specificity of anti-phosphotyrosine antibodies, proteins were dephosphorylated prior to gel electrophoresis by incubating the samples for 20 min at 30°C with 6 µL (400 units) lambda phosphatase (Santa Cruz, CA) in 50 mM Tris Buffer pH 7.5, 0.1 mM Na_2_EDTA, 5 mM dithiothreitol, 2 mM MnCl_2_.

### Membrane protein cluster separation by size exclusion chromatography

AE1 was labeled with eosine maleimide and red cell membranes were fractionated as previously described [34]. With minor modifications, 1 ml membranes were solubilized in 2 ml extraction buffer (10 mM Hepes, 130 mM NaCl, 10 mM *N*-ethylmaleimide, 1 mM EDTA, 1 mM PMSF, 1% triton-X100, pH 7.4), gently shaken for 10 min at 20°C and centrifuged at 13000 rpm for 5 min in an Eppendorf microfuge. The supernatant was applied to a 40×1 cm column filled with Sepharose CL-6B equilibrated with a solution containing 10 mM Hepes, 50 mM NaCl, 0.1% triton-X100 (pH 7.4), at a flow rate of 1 ml/min. Constant flow was maintained using an HPLC pump. The effluent was collected in 1 ml fractions. Eosine maleimide fluorescence was quantified as described [18] and, to measure the percentages of clustered AE1, the fluorescence measured in the high molecular weight fraction was expressed as a percentage of the total fluorescence eluted from the columns. The high molecular weight fraction was concentrated 10 fold and analysed for immunoblotting with anti-AE1, anti-phosphotyrosine and anti-Syk as described above.

### Preparation of cells for immunofluorescence

Control and G6PD deficient RBCs were pelleted and washed twice in PBS 1× containing 5 mM glucose and then fixed for 5 min in 0.5% acrolein in PBS. Cells were rinsed three times then permeabilized in PBS containing 0.1 M glycine (rinsing buffer) plus 0.1% Triton X-100 for 5 min and again rinsed 3× in rinsing buffer. To ensure complete neutralization of unreacted aldehydes, the cells were then incubated in rinsing buffer at room temperature for 30 min. After incubation, all nonspecific binding was blocked by incubation again for 60 min in blocking buffer (PBS containing 0.05 mM glycine, 0.2% fish skin gelatin and 0.05% sodium azide). Staining of fixed, permeabilized RBCs was performed by using specific antibodies diluted in blocking buffer. After labeling, resuspended RBCs were allowed to attach to cover slips coated with polylysine, and the cover slips were mounted by using Aqua-Mount (Lerner Laboratories, New Haven, CT). The auto-fluorescence of hemichromes was visualized by exciting at 488 nm and observing their emission in the 630–750 nm range. Samples were imaged with a Bio-Rad MRC1024 (Bio-Rad) confocal microscope equipped with a 60×1.4 numerical aperture oil immersion lens.

### Assay of hemichromes

Hemichromes were quantified by measuring heme absorbance at 560, 577 and 630 nm [11] and expressed as nmoles/mL of solubilised membranes.

### Assay of glutathione

GSH estimations were performed using 5,5′-dithiobis(2-nitrobenzoic acid) (DTNB) [44].
